# P-1334. Monotherapy versus Combination Antimicrobial Therapy for Pseudomonas aeruginosa Left Ventricular Assist Device Infections

**DOI:** 10.1093/ofid/ofaf695.1522

**Published:** 2026-01-11

**Authors:** Mayyadah Alabdely, Patricia Bartley, Jona Banzon, Nabin K Shrestha

**Affiliations:** Cleveland Clinic Foundation, Cleveland, OH; Cleveland Clinic, Cleveland, OH; Cleveland Clinic, Cleveland, OH; Cleveland Clinic, Cleveland, OH

## Abstract

**Background:**

Treatment of *Pseudomonas aeruginosa* left ventricular assist device (LVAD) infections is particularly challenging because of the pathogen’s virulence and propensity for development of resistance while on treatment. Our practice has begun to consider the possibility that combination therapy, as opposed to monotherapy, may limit the development of antimicrobial resistance. The purpose of this study was to compare the outcome of resistance development between monotherapy and combination therapy for *P. aeruginosa* LVAD infections.

**Figure:**
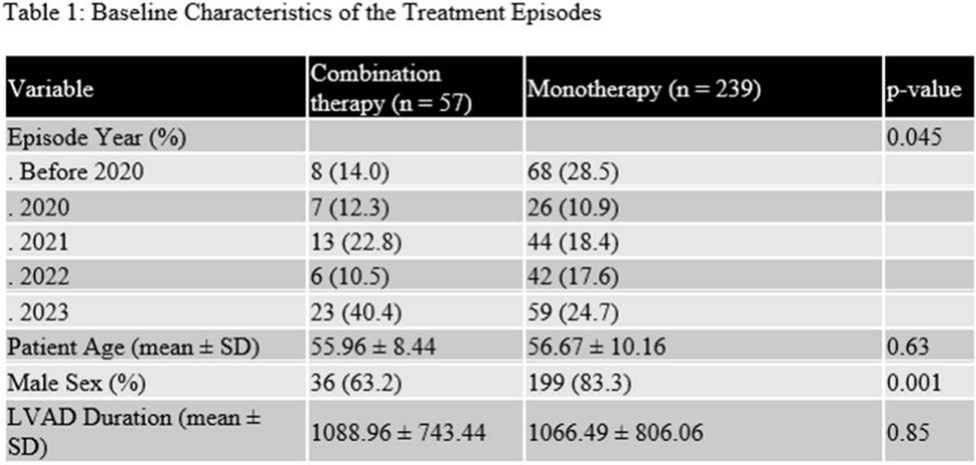

**Figure:**
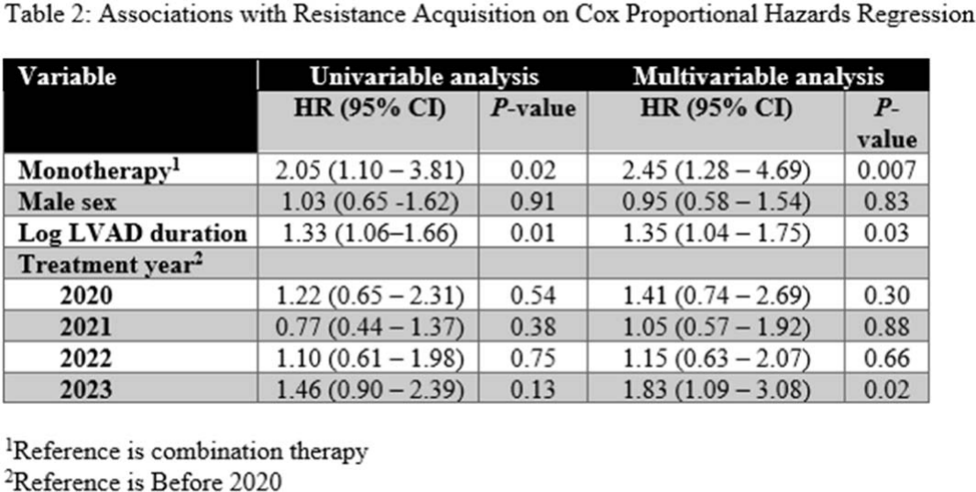

**Methods:**

This was a retrospective cohort study of episodes of LVAD infections due to *Pseudomonas aeruginosa* in adult patients (age >18) treated between Jan 1, 2011 and December 31, 2023. The primary outcome was time to resistance acquisition, defined as a change in susceptibility interpretation or a four-fold increase in MIC to a treatment agent. Cox proportional hazards regression was performed to identify factors associated with resistance acquisition. Covariates included treatment strategy (monotherapy vs. combination therapy), age, sex, duration of LVAD support, and the episode year.

**Results:**

A total of 296 treatment episodes of *P. aeruginosa* LVAD infections were identified in 76 patients (53 male, 19 female). During these treatment episodes, the mean patient age was 57 years (SD 10), and mean LVAD duration was 1071 days (SD 793). Of these treatment episodes 57 (19%) consisted of combination therapy. Table 1 compares baseline characteristics of these treatment episodes. In a multivariable Cox proportional hazards regression model, monotherapy was associated with a significantly higher risk of resistance acquisition (HR 2.45, 95% CI 1.28-4.69, *p* = 0.007) ( Table 2).

**Conclusion:**

In patients with *Pseudomonas aeruginosa* LVAD infections, monotherapy was associated with a significantly higher risk of resistance acquisition compared to combination therapy.

**Disclosures:**

All Authors: No reported disclosures

